# P-1670. Digital Five-Step Tool Enhances Standard Perioperative Prophylaxis by Delabeling Surgical Patients from Allergy to Beta-Lactam Antibiotics

**DOI:** 10.1093/ofid/ofae631.1836

**Published:** 2025-01-29

**Authors:** Daniel Röder, Kathrin Eichhorn, Patrick Meybohm, Johanna Stoevesandt, Güzin Surat

**Affiliations:** University Hospital Würzburg, Würzburg, Bayern, Germany; University Hospital Würzburg, Würzburg, Bayern, Germany; Department of Anaesthesiology, Intensive Care, Emergency and Pain Medicine, University Hospital Würzburg, Würzburg, Würzburg, Bayern, Germany; University Hospital Würzburg, Würzburg, Bayern, Germany; University Hospital Würzburg, Würzburg, Bayern, Germany

## Abstract

**Background:**

Up to 90% of patient-reported allergy to one or more beta-lactam antibiotics (BLA) are misdiagnosed, underscoring the widespread issue of incorrect allergy identification. The Infectious Diseases Society of America (IDSA) guidelines and local Antimicrobial Stewardship Programs advocate for the delabeling of self-reported BLA allergies in preoperative settings to facilitate the administration of BLAs, the standard for perioperative antibiotic prophylaxis (PAP). This study evaluates the safety of a novel, digitally-based questionnaire designed to delabel surgical patients from BLA allergies, promoting the use of standard PAP.

Digital five-step tool to delabel surgical patients from allergy to beta-lactam antibiotics

**Figure:**
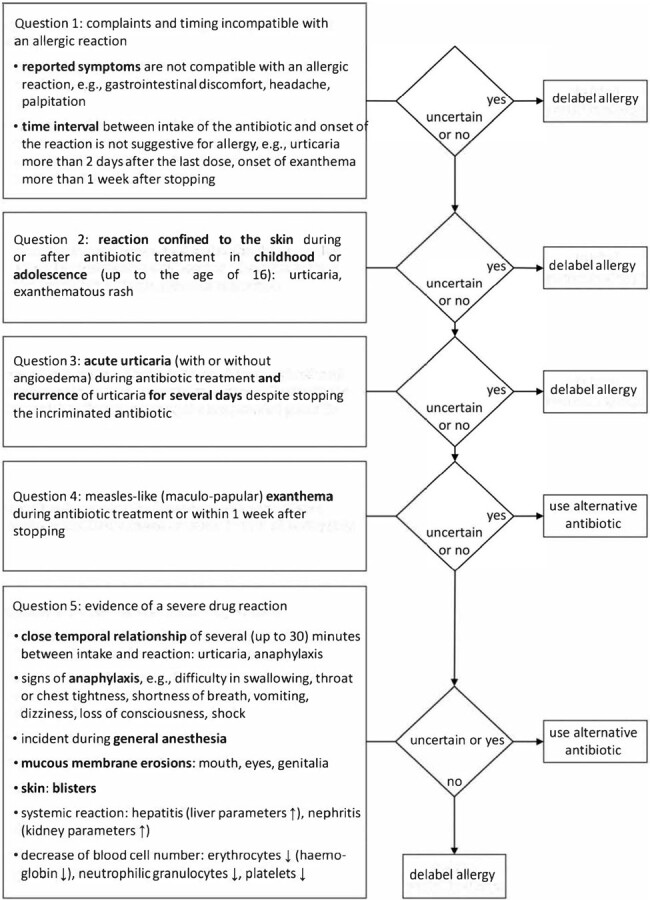

Workflow of the digital delabeling tool as published by Schrüfer (2022) Allergy, Asthma & Clinical Immunology, 18(1), 26.

**Methods:**

A single-center retrospective observational study was conducted from September 2020 to October 2022, analyzing surgical patients who reported BLA allergies during preoperative assessments. A digital tool integrated into the patients’ electronic health records assessed the risk of a genuine allergy using five specific questions and then provided recommendations for or against the use of standard PAP (Figure 1). Subsequent allergic reactions to PAP were monitored for 24 hours.

**Results:**

Of the 983 patients reporting BLA allergy, 322 (32.7%) did not require PAP. Among the remaining 661 patients, the questionnaire targeted 420 (63.5%) for delabeling. Within this group, 262 patients (62.4%) received standard BLAs and reported two allergic reactions (0.8%), while 158 (37.6%) were administered alternative antibiotics, experiencing two allergic reactions (1.3%). The tool’s negative predictive value was 99.2%. Conversely, of the 241 patients advised to maintain the BLA allergy label, 197 (81.7%) received alternative antibiotics with four allergic reactions (2.0%), and 44 (18.3%) were administered BLAs contrary to the tool’s advice, yet experienced no allergic reactions.

**Conclusion:**

The use of a novel, digitally-based five-step questionnaire in the preoperative setting successfully delabeled reported BLA allergies in over 60% of patients. This tool proved straightforward and safe, substantially enhancing the use of standard first-line antibiotics and thereby adhering to best practice guidelines.

**Disclosures:**

**Daniel Röder, MBA**, Biotest AG: Honoraria

